# Prolyl-4-Hydroxylase 3 (PHD3) Expression Is Downregulated during Epithelial-to-Mesenchymal Transition

**DOI:** 10.1371/journal.pone.0083021

**Published:** 2013-12-18

**Authors:** Trenton L. Place, Jones T. Nauseef, Maina K. Peterson, Michael D. Henry, James J. Mezhir, Frederick E. Domann

**Affiliations:** 1 Molecular and Cellular Biology Program, The University of Iowa, Iowa City, Iowa, United States of America; 2 Department of Radiation Oncology, The University of Iowa, Iowa City, Iowa, United States of America; 3 Department of Molecular Physiology and Biophysics, The University of Iowa, Iowa City, Iowa, United States of America; 4 Department of Anatomy and Cell Biology, The University of Iowa, Iowa City, Iowa, United States of America; 5 Department of Surgery, The University of Iowa, Iowa City, Iowa, United States of America; AMS Biotechnology, United Kingdom

## Abstract

Prolyl-4-hydroxylation by the intracellular prolyl-4-hydroxylase enzymes (PHD1-3) serves as a master regulator of environmental oxygen sensing. The activity of these enzymes is tightly tied to tumorigenesis, as they regulate cell metabolism and angiogenesis through their control of hypoxia-inducible factor (HIF) stability. PHD3 specifically, is gaining attention for its broad function and rapidly accumulating array of non-HIF target proteins. Data from several recent studies suggest a role for PHD3 in the regulation of cell morphology and cell migration. In this study, we aimed to investigate this role by closely examining the relationship between PHD3 expression and epithelial-to-mesenchymal transition (EMT); a transcriptional program that plays a major role in controlling cell morphology and migratory capacity. Using human pancreatic ductal adenocarcinoma (PDA) cell lines and Madin-Darby Canine Kidney (MDCK) cells, we examined the correlation between several markers of EMT and PHD3 expression. We demonstrated that loss of PHD3 expression in PDA cell lines is highly correlated with a mesenchymal-like morphology and an increase in cell migratory capacity. We also found that induction of EMT in MDCK cells resulted in the specific downregulation of PHD3, whereas the expression of the other HIF-PHD enzymes was not affected. The results of this study clearly support a model by which the basal expression and hypoxic induction of PHD3 is suppressed by the EMT transcriptional program. This may be a novel mechanism by which migratory or metastasizing cells alter signaling through specific pathways that are sensitive to regulation by O_2_. The identification of downstream pathways that are affected by the suppression of PHD3 expression during EMT may provide important insight into the crosstalk between O_2_ and the migratory and metastatic potential of tumor cells.

## Introduction

Cell migration is a highly choreographed process that involves crosstalk between plasma membrane receptors, signaling proteins, and the actin cytoskeleton[1]. Cell migration is typically a characteristic of mesenchymal cells. However, epithelial cells are also able to become motile through a process termed epithelial-to-mesenchymal transition (EMT)[2]. EMT occurs in many physiological processes, including development, wound-healing, and cancer[2]. It is recognized that many differences exist in the EMT phenotype depending on the physiological setting involved. However, all types of EMT generally involve the activity of SNAIL, Zeb, or Twist family members[2]. These are transcriptional repressors that bind to specific sequences in the promoters of genes involved in epithelial polarity and suppress their transcription[2]. The epithelial cell-cell adhesion molecule, E-cadherin (*CDH1*), is a classical target of these repressors[2]. During EMT, downregulation of E-cadherin allows detachment of epithelial cells from their neighbors. Additionally, genes involved in cytoskeletal rearrangement and extracellular matrix (ECM) degradation are also controlled by EMT-mediated transcriptional reprogramming[2]. Taken together, the process of EMT ultimately promotes cell motility and invasion, which is important in development, wound healing, tissue regeneration, and cancer metastasis.

In epithelial tumors, EMT is thought to be induced by several mechanisms including cytokine and growth factors (e.g. TGF-β), as well as by hypoxia[2], [3]. Cytokines and growth factors, which are produced by the tumor stroma, activate signaling pathways that lead to the upregulation of *SNAIL, Zeb*, and *Twist* [2], [3]. Similarly, hypoxia-induced EMT also appears to involve features of cytokine and growth factor-induced EMT, however, it also includes interplay with numerous other signaling pathways[3]. Thus, the role of hypoxia in EMT and cell migration still remains ill defined.

The signaling effects of hypoxia on the cell are largely mediated by the oxygen-sensing activity of a small family of intracellular prolyl-4-hydroxylases (PHD1, 2 and 3)[4]. PHDs are dependent on molecular oxygen (O_2_) to enzymatically place a hydroxyl group on specific proline residues on target proteins[4]. The canonical PHD-target is the hypoxia-inducible transcription factor (HIF), which controls the expression of numerous genes involved in the adaption of cells to hypoxia, as well as the EMT transcriptional repressor Twist1[5], [6].

The PHD enzymes also have HIF-independent effects. For example, PHD2 expression appears to promote motility, possibly through direct PHD2-mediated effects on the cell cytoskeleton[7]. PHD3 appears to have an even broader spectrum of non-HIF target proteins[4]. In macrophages, knockout of PHD3 results in cells that are more motile than controls[8]. When PHD3 is knocked-down in cancer cells, cell migration is also potentiated[9]. This suggests that the loss of PHD3 is related to cell migration, but further insight has yet to be put forth.

Previous work in our laboratory has focused on the expression of PHD3 in cancer cells. We have found that numerous cancer cell lines silence PHD3 expression. This silencing, which can occur through DNA methylation of the PHD3 promoter, does not appear to effect HIF activity[10]. Thus we hypothesized that PHD3 silencing in cancer cells instead may be related to EMT, which is known to cause to the downregulation of numerous genes[11], [12].

To understand whether PHD3 is directly involved in cell motility, we examined cell morphology and migratory capacity in pancreatic ductal adenocarcinoma (PDA) cells that possessed either over-expressed or knocked-down levels of PHD3. Furthermore, we examined the effect of the EMT transcriptional program on PHD3 expression. We used the Madin-Darby Canine Kidney (MDCK) cell line, a well-established model of EMT, to study the effects of EMT induction on PHD3 expression in epithelial cells [13]. We found that knock-down of PHD3 is associated with an increased migratory capacity in PDA cells. Furthermore, our results validate PHD3 as a novel gene that is targeted for downregulation following the induction of EMT. Our data further unravel the complex interaction between hypoxia-response proteins and cell migration, supporting other data that demonstrate PHD3 as an important player in tumor cell migration and metastasis.

## Methods

### Cell Culture

Normal Human Fibroblast (NHF-1) cells obtained from the Coriell Cell Repositories (AG01522) were a gift from Dr. Prabhat Goswami's Laboratory (University of Iowa). MiaPaca2, Panc1, BxPC3 (primary pancreatic cancer cell lines), CAPAN1 (pancreatic cancer liver metastasis cell line) and MDCK-parental (NBL-2) cells were purchased from ATCC. MDCK, MiaPaca2, Panc1, and BxPC3 cells were grown in DMEM high glucose medium with 10% FBS (Atlanta Biologicals) and supplemented with Pen/Strep. For glucose deprivation, DMEM without glucose, glutamine, pyruvate or FBS was utilized (Gibco). All cell lines were routinely maintained at 37°C in a humidified atmosphere with 5% CO_2_. Cells were maintained in log phase growth with fresh media replacement at least every three days. Routine subculture was performed by detaching cells with TrypLE Express (Invitrogen). Hypoxic experiments were performed using a static hypoxia chamber flooded with a gas mixture containing 5% CO2, ∼94% Nitrogen and either 1% or 0.1% Oxygen.

### Quantitative Real-Time PCR

For RNA isolation, cells were dissociated in Trizol reagent on the tissue culture plate following removal of cell culture media. Reverse transcription was carried out on 1 ug of total RNA with High-Capacity cDNA Archive Kit with random hexamers (Applied Biosystems, Foster City, CA). Real-time PCR primers were designed using the Roche Universal Probe Library Assay Design with default settings. When possible, primer sets were designed to span an intron. Primer oligo sets were ordered through Integrated DNA Technologies (IDT, Coralville, Iowa) with standard desalting. The primer sequences utilized for SYBR Green quantitative real-time PCR can be found in **Table S1**. For human PHD3, Taqman primer probe set Hs00222966_m1 was used (Life Technologies, Grand Island, New York). The quantitative real-time PCR was set up as follows: 10 ng of cDNA was used as template for each real-time PCR reaction (10 µl reaction volume). When SYBR Green was used, primers were used at 0.5 µM final concentration. For all reactions, the DNA polymerase was activated by heat at 95°C for 10 min followed by 40 cycles, denaturing at 95°C for 15 s, annealing and elongating at 60°C for 1 min. Data were collected with ABI PRISM 7000 sequence detection system. Data were analyzed using the ΔΔCt method.

### Western Blot Analysis

Cells were immediately washed with ice cold phosphate-buffered saline (pH 7.4). Cells were lysed on the plate in RIPA cell-lysis buffer (50 mM Tris pH 8.0, 150 mM NaCl, 0.1% SDS, 0.5% Na Deoxycholate, 1% TX-100) plus 1 mM NaF, 10 mM NaVO_4_, 10 mM PMSF, and 1/100 protease inhibitor cocktail (Sigma) then sonicated for 30 seconds at 50% amp. SDS-polyacrylamide gels (12%, PHD3; 7% HIF1, HIF2) were used for protein electrophoresis. Proteins were electrotransferred onto nitrocellulose membranes and blocked with 5% milk in PBS-T for 1 hour. Western blots for PHD3 on human samples were blocked in 3%milk + 2% Pro Performance® AMP Amplified 100% Whey Protein (GNC Corporation, Pittsburgh, PA) in PBS-T for 1 hour. All membranes were then blotted in primary and secondary antibodies with 0.5% milk in PBS-T follows: Anti HIF-1α (Abcam, Cambridge, MA) 1∶500 overnight at 4°C. Anti PHD3, NB100-139, Anti PHD2 NB100-137 anti HIF-2α antibodies (Novus Biologicals, Littleton, CO) were used at 1∶500 overnight at 4°C. Equal protein loading was confirmed on all immunoblots using human beta-tubulin monoclonal antibody (E7 – UIowa Hybridoma Facility) at a dilution 1∶2000. Goat anti-rabbit or anti-mouse HRP-conjugated IgG (BD Transduction Laboratories, San Diego, CA) was used as a secondary antibody (1∶20,000). Bands were resolved by chemiluminescence with Super Signal West Pico Chemiluminescent Reagent (Pierce, Rockford, IL) using film.

### Sodium Bisulfite Sequencing

Genomic DNA was extracted with the use of the DNeasy Tissue Kit (Qiagen, Valencia, CA), and sodium bisulfite conversion was performed with the use of the EZ DNA Methylation Kit (Zymo Research Corporation, Orange, CA). Methprimer (www.urogene.org) was used to design 2 sets of primers complementary non-CpG containing sequence stretches in the dog *PHD3* promoter. Nested PCR amplification on converted DNA used the following primers: outside forward (pTP-154), outside reverse (pTP-155), inside forward (pTP-156), and inside reverse (pTP-157). For primer sequences used see **Table S1**. The resulting PCR products were gel-extracted with the use of the Qiagen Gel Extraction Kit, and cloned with the CloneJET PCR Cloning Kit (Thermo Scientific). Plasmids were transformed into DH5α E. *coli* and plated on Ampicillin-Agar plates. Clones were picked and plasmid DNA was extracted from overnight cultures using a QiaPrep Spin Plasmid Miniprep Kit (Qiagen). Sequencing was performed by the sequencing core facility maintained by the University of Iowa and results were tabulated for methylation status of each of the CpGs contained within the amplicon.

### Vectors

For stable PHD3 expression, full-length PHD3 or PHD3H196A cDNA sequences were cloned into the pQCXIP retroviral packaging vector. pQCXIP constructs, along with a plasmid containing the VSVg envelope protein were transfected into 90% confluent GP293 cells on 60 mm dishes using Lipofectamine 2000 according to the manufacture's protocol. Transfection reagent was removed after 6 hours and replaced with DMEM containing 10% FBS. Twelve hours later, the media was replaced with 3 ml DMEM containing 20% FBS. Every six hours, viral supernatant (media) was collected and filtered through a .45 µM low-protein binding syringe filter. Viral supernatant was then added directly to cell lines (at 75% confluency in 60 mm dishes) and allowed to transduce cells for 8 hours. Media was then replaced with fresh growth medium. 36 hours following transduction, cells were split into 10 cm dishes in 9 ml DMEM + 1 µg/ml Puromycin (BxPC3, MiaPaca2) or 3 µg/ml Puromycin (MDCK cell lines).

For stable PHD3 knockdown, pLKO.1 Lentiviral shRNA constructs were purchased from the RNAi consortium (TRC-Hs1.0) through Openbiosystems (Clone#-TRCN0000001046, TRCN0000001047, TRCN0000001048, TRCN0000001049, TRCN0000001050). Constructs are abbreviated hereafter by the last two numbers (e.g. 46, 47, 48, 49, 50). For stable PHD3 knockdowns, pLKO constructs were co-transfected along with VSVg and pCMV-dR-8.91 into the 293T packaging cell line using Lipofectamine 2000. Subsequent virus collection and transduction protocol was identical to that described for pQCXIP-retrovirus (above). After screening of all knockdown constructs, #48 consistently produced the most effective knockdown of PHD3 and was therefore used in experiments

### Immunofluorescence

Cells were washed in cold HBSS + Mg + Ca and fixed in freshly prepared 4% paraformaldehyde pH 7.2 for 20′ at 4°C. Cells were permeabilized in 0.25% TX-100 in PBS for 5′ at room temperature (RT) then blocked in PBS + 10% FBS (filtered) for 30′ at RT. Cells were then incubated in anti-dog-E-cadherin antibody (1∶200 rr1-University of Iowa Hybridoma Facility, Iowa City, USA) for 30′ RT, washed 3x in PBS then labeled with goat anti-mouse-Alexa® Fluor-488 (1∶200) and -568 (1∶200) (Life Technologies, Carlsbad, CA, USA) for 30′ RT. Cells were visualized in a Zeiss 510 confocal microscope.

### Scratch Assay

2 million BxPC3 cell were seeded into 60 mm dishes. The following day, dishes were placed in a hypoxia chamber containing 1% O_2_, 5% C0_2_, and 94% N_2_ for 24 hours. Plates were removed from hypoxia and a reference line was drawn on the bottom of the plate, followed by perpendicular scratches to the cell side of the dish with the tip of a 5 ml stripette. ImageJ was utilized to find the inside area of each scratch. Migration speed was determined by the following formula. Migration Speed  =  change in inside area of scratch/(time x scratch height).

### Flow Cytometry

Cells assayed by flow cytometry were released from adherent culture using Versene (Gibco) for ∼15 minutes, or until all cells had released from the plate. Versene was then diluted with complete media and the cell suspension was centrifuged at 1200 rcf for 5 minutes, after which the supernatant was aspirated and cell pellets resuspended in complete cell culture media. Cells were counted using a hemacytometer, washed in FACS buffer (0.1% NaAzide, 1% BSA in 1X PBS), and 200,000 cells were suspended in antibody in FACS buffer at a final staining volume of 50 µL. Primary antibody treatments were either anti-dog-E-cadherin (mIgG1, rr1, 1∶200, Developmental Studies Hybridoma Bank, University of Iowa), anti-human-E-cadherin (mIgG1, 1∶200, R&D Systems) isotype control (1∶200, Sigma) for 1 hour on ice. Samples were then washed three times in 1 mL cold FACS buffer. Secondary staining was performed using mIgG DyLight 488 (Jackson ImmunoResearch) (dog samples), or goat anti mouse IgG-FITC (Millipore) (human samples) at 1∶200 in 50 µL staining volume for 1 hour on ice protected from light. Periodic gentle mixing during both primary and secondary incubations was employed. Samples were then washed as before, and suspended in a final volume of 250 µL FACS buffer with the addition of Hoechst 33258 at a final concentration of 16 µL/ml. Samples were assayed on a Becton Dickinson LSR with violet laser. All cells quantified excluded Hoechst 33258. Data analysis was performed using FlowJo software.

### Immunoprecipitation

Subconfluent cells were grown in 10 cm dishes and placed in normoxic or hypoxic conditions for 24 hours. Cells were washed twice under hypoxia with cold HBSS + Mg +Ca, that had been pre-equilibrated in the hypoxia chamber for 24 hours. Lysis was carried out on the plates under hypoxia in NP-40 lysis buffer containing 50 mM Tris-HCl pH 7.5, 150 mM NaCl,?5 mM EDTA,?0.5% NP-40, protease inhibitor cocktail, PMSF, NaF, and NaVO_4_. 50 µl protein A Dynabeads were blocked in 10% FBS in PBS and prebound with 2.5 µg PHD3 antibody (Novus, NB100-139) or HA.11 (Covance, AB641). 1 mg of whole cell lysate in 250 µl lysis buffer was applied to beads for 1 hr at 4°C. Beads were washed twice in IP wash buffer (50 mM Tris-HCl pH 7.5, 150 mM NaCl, 5 mM EDTA,0.25% NP-40), and protein was eluted by boiling beads in Laemmli buffer for 2 minutes.

## Results

### PHD3 knockdown in BxPC3 cells results in a cell-cell adhesion defect and increased migratory capacity

**Figure 1:**
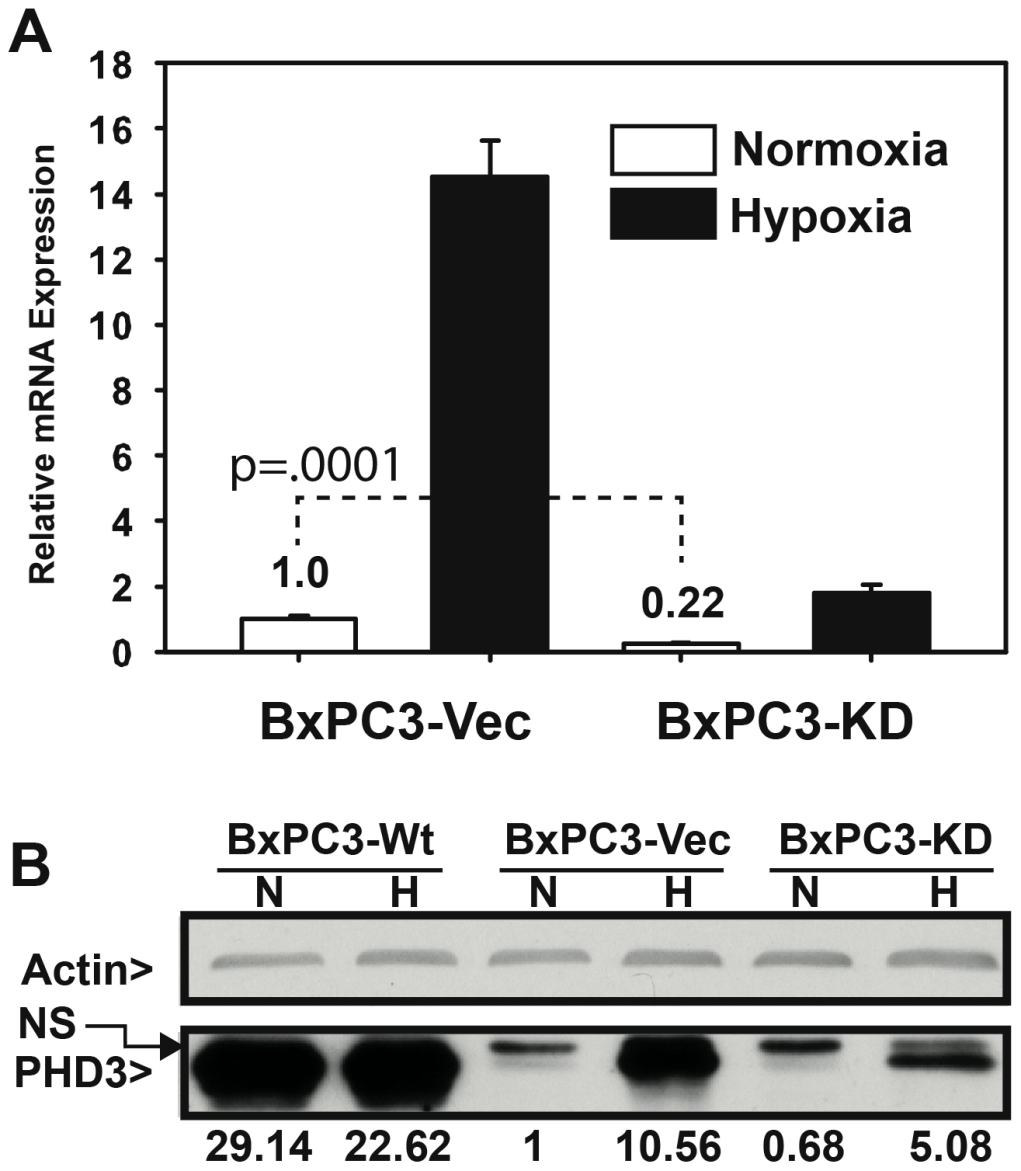
PHD3 expression in BxPC3 cells. BxPC3 cells stably transduced with retrovirus containing PHD3Wt (BxPC3-Wt), Vector (BxPC3-Vec) or anti-PHD3 shRNA (BxPC3-KD) were harvested for RNA and protein following 24 hours exposure to normoxia (21% O_2_) or hypoxia (1% O_2_). (A) PHD3 mRNA expression was determined by qRT-PCR in BxPC3-Vec and BxPC3-KD cells. All samples were normalized to 18S rRNA and graphed as expression relative to BxPC3-Vec Normoxia (lane 1). n = 3, error bars  = 1 S.D. p-value represents Student's 2-tailed, type 2 t-test comparison (B) Whole cell lysate from BxPC3-Wt, BxPC3-Vec and BxPC3-KD cells following 24 hours exposure to normoxia (N) or hypoxia (H) was resolved by SDS-PAGE and blotted for actin (top) and PHD3 (bottom). PHD3 band intensity was quantified relative to actin in each lane and then normalized to BxPC3-Vec (N). Relative band intensity is indicated below the figure. NS  =  non-specific band running just above PHD3 band. Data is representative of >3 biological replicates.

**Figure 2:**
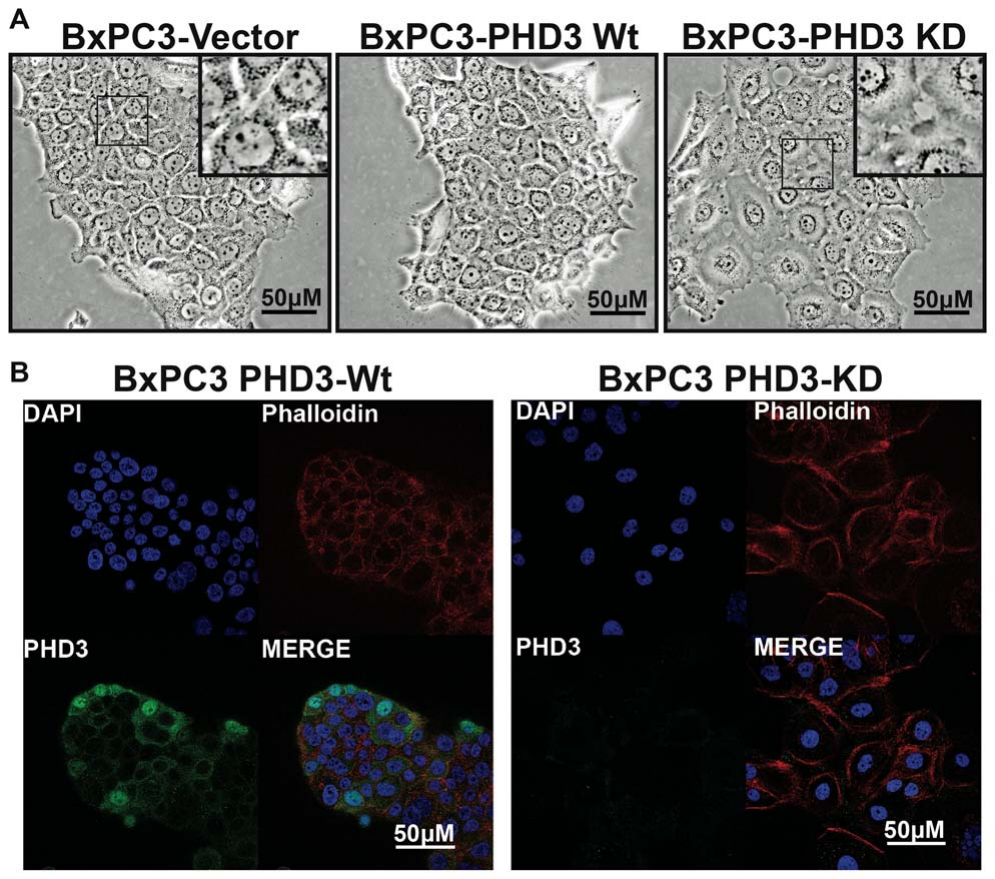
PHD3 knockdown affects cell-cell adhesion and actin cytoskeletal morphology. (A) BxPC3 cells stably transduced with retrovirus containing PHD3Wt (BxPC3-Wt), Vector (BxPC3-Vec) or anti-PHD3 shRNA (BxPC3-KD) were grown on tissue culture dishes for 48 hours and live cells were photographed at 20× using phase contrast microscopy. (B) Stable BxPC3-Wt and BxPC3-KD cells grown on glass coverslips were labeled with PHD3 antibody (green) and phalloidin (red) and DAPI (blue) and photographed at 20× using a Zeiss 510 confocal microscope.

Previous studies have shown a negative correlation between PHD3 expression and cell invasiveness through Matrigel™, indicating that PHD3 loss may promote mesenchymal and/or metastatic properties in cancer cells[9]. To validate these findings, we performed stable knockdown using PHD3-specific shRNA (BxPC3-KD) and overexpression of PHD3 (BxPC3-Wt) in the PDA line BxPC3. This cell line was isolated from a primary human pancreatic adenocarcinoma and has an “epithelial-like” phenotype in normal cell culture [14]. In BxPC3-KD cells, basal PHD3 mRNA expression was reduced by >75% and hypoxia-inducible expression by >98% (**Figure 1A**). Under normoxic conditions, detection of PHD3 protein was difficult due to limits of detection of the antibody. However, under hypoxic conditions, it was clear that BxPC3-KD cells had a significant reduction in PHD3 protein levels compared to the vector control (BxPC3-Vec) stable cell line (**Figure 1B**). The effects of PHD3 knockdown on cell morphology were also strikingly apparent. BxPC3-KD cells appeared flattened, more scattered, and appeared to have difficulty forming cell-cell junctions when compared to BxPC3-Wt and -Vec cells (**Figure 2**). When we labeled these cells with fluorescent-phalloidin (phalloidin-Alexa® Fluor 568), cortical actin rings in PHD3-KD cells were easily distinguishable from neighboring cells and appeared less organized. This is in contrast to BxPC3-Wt and BxPC3-Vec cells, in which actin appeared more tightly associated with cell-cell junctions (**Figure 2B**) (data not shown for BxPC3–Vec cells). Although cell-cell junctions in BxPC3-KD cells were different in appearance when compared to -WT and -Vec cells, there were no differences in E-cadherin surface expression as could be determined by flow cytometry (**Figure S1**).

**Figure 3:**
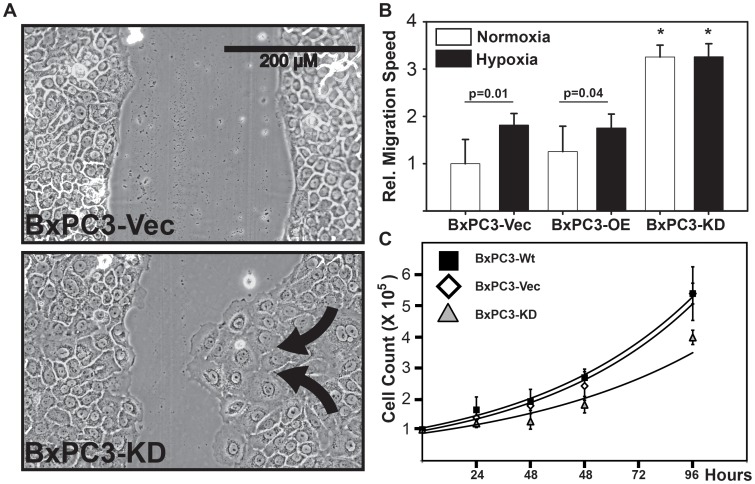
PHD3 knockdown increases the migratory capacity of cells. BxPC3 cells stably transduced with retrovirus containing PHD3Wt (BxPC3-Wt), Vector (BxPC3-Vec) or anti-PHD3 shRNA (BxPC3-KD) were seeded at confluence in 60 mm tissue culture dishes and allowed to adhere for 24 hours. (A) Scratches were made using a 5 ml stripette and photographed at 0 hr and 24 hr under normoxia (21% O_2_) or hypoxia (1% O_2_). Arrows highlight the directional migration of cells (B) Migration speed for each cell line was normalized to BxPC3-Vec normoxia. Data is representative of 3 independent biological replicates and 8 scratches each. Error bars  = 1 S.D. p-value represents Student's 2-tailed, type 2 t-test comparison. * samples are significantly different p<.01 than all other samples. (C) Live cells were counted using trypan blue exclusion at the indicated time points. Lines represent best fit for the data.

We next evaluated whether the cell-cell attachment defect in the BxPC3-PHD3 KD cells was correlated with an increased migratory capacity. In a scratch assay, PHD3-KD cells appeared to actively migrate into the scratch. This was evident by numerous extended lamellipodia (**Figure 3A**). BxPC3-Vec and Wt-expressing cells did not extend lamellipodia to this extent. Furthermore, quantification of migration speed into the scratch demonstrated that BxPC3-KD cells migrated nearly 3 fold faster than BxPC3-Vec and PHD3-Wt overexpressing cells. (**Figure 3B**), supporting a previous report by Su et al.[9]. Interestingly, the increased migration speed of the BxPC3-KD cells was not further induced by hypoxia, suggesting that migration was already maximally uninhibited in these cells. The increased migration speed into the scratch also did not appear to be due to an increase in proliferation of the BxPC3-KD cells, as BxPC3-KD cells proliferate at a noticeably slower rate. This observation was quantified by growth curve, where BxPC3-KD cell have a doubling time that is 8 hours slower than BxPC3-Vec or Wt cells (**Figure 3C**).

### PHD3 silencing is associated with a mesenchymal-like morphology in human pancreatic cancer cell lines

**Figure 4:**
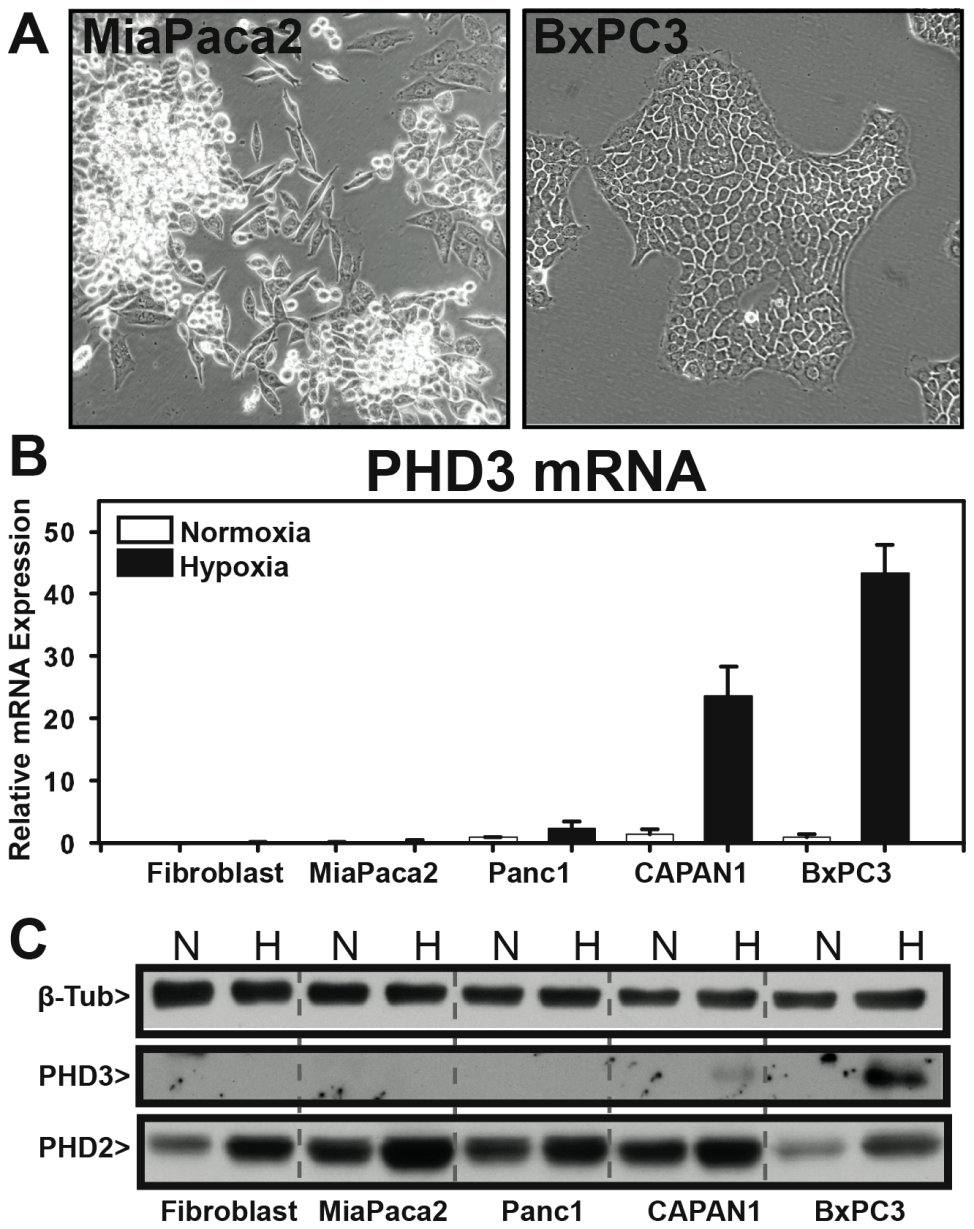
PHD3 expression correlates with a mesenchymal-like morphology in pancreatic ductal adenocarcinoma cell lines. NHF-1 (Fibroblast) MiaPaca2, Panc1, CAPAN1 and BxPC3 cells were harvested for RNA and protein following 24 hours exposure to normoxia (21% O_2_) or hypoxia (1% O_2_). (A) Phase-contrast images at 10× magnification were taken of MiaPaca2 (mesenchymal-like) morphology and BxPC3 cells (differentiated, epithelial morphology) under normoxic conditions. (B) PHD3 mRNA expression was determined by qRT-PCR and graphed relative to BxPC3 in normoxia. All samples were normalized to 18S rRNA and graphed as expression relative to BxPC3-Vec Normoxia (lane 1). n = 3, error bars  = 1 S.D. (C) Whole cell lysate was resolved by SDS-PAGE and blotted for β-tubulin PHD3 and PHD2. N = normoxia, H = hypoxia (1% O_2_).

Since PHD3 knockdown in BxPC3 cells resulted in a cell-cell adhesion defect, we hypothesized that natural variation of PHD3 expression in cell lines should directly correlate with their cell-cell adhesion characteristics. To test this hypothesis, we chose a panel of three pancreatic ductal adenocarcinoma cell lines (CAPAN1, Panc1 and MiaPaca2) that displayed a range of morphologies in cell culture. CAPAN1 and BxPC3 cells are relatively “epithelial-like”, growing in sheets of tightly connected cells. MiaPaca2 and Panc1 cells, on the other hand, are much more “mesenchymal-like” in appearance, with fewer cell-cell junctions and a more scattered distribution in standard culture (**Figure 4A**).

We analyzed PHD3 mRNA and protein expression under normoxic (21% O_2_) and hypoxic (1% O_2_) conditions in each of these PDA cell lines. Furthermore, we utilized the NHF-1 fibroblast cell line as a mesenchymal cell type for direct comparison. Similar to previous studies that examined PHD3 expression in cancer cell lines, we found a wide range of PHD3 expression in our panel of PDA cells [10], [15]. The mesenchymal-like MiaPaca2 cell line had nearly undetectable levels of PHD3 mRNA and protein expression under normoxic conditions, and was not upregulated by exposure hypoxic conditions. This absence of PHD3 expression was mirrored in the fibroblast cell line, NHF-1. In stark contrast, the epithelial-like CAPAN1 and BxPC3 cell lines expressed detectable PHD3 mRNA under normoxic conditions, with a 17 fold and 43-fold increase in PHD3 mRNA expression under hypoxic conditions, respectively. Although PHD3 protein was difficult to detect with our antibody in any of the cells under normoxic conditions, it became easily detected following exposure to hypoxia in BxPC3 and CAPAN1 (**Figure 4B and C**).

Overall, these results show a strong correlation between loss of PHD3 expression and a mesenchymal-like morphology in cell culture. This suggested to us that PHD3 expression is connected to epithelial and not mesenchymal cell differentiation. In pancreatic cancer, SNAIL has been implicated as a key driver of EMT [16]. Therefore, we asked if there was a correlation between *PHD3* gene silencing and markers of EMT (loss of E-cadherin and upregulation of SNAIL) in our four PDA cell lines.

**Figure 5:**
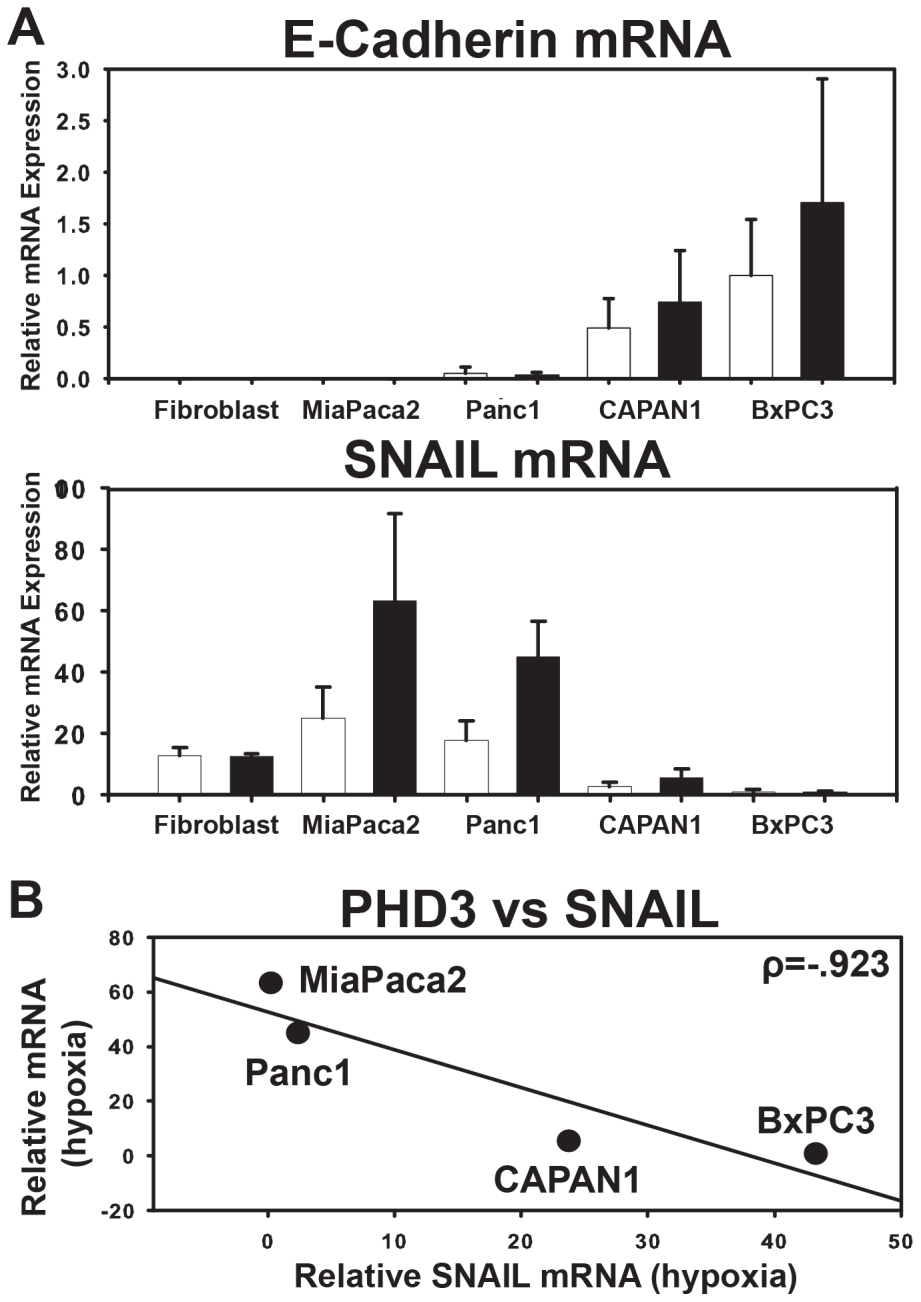
E-cadherin and PHD3 expression are inversely correlated with SNAIL expression. NHF-1 (fibroblast), MiaPaca2, Panc1, CAPAN1, and BxPC3 cells were harvested for RNA following 24 hours exposure to normoxia (21% O_2_) or hypoxia (1% O_2_). (A) E-cadherin and SNAIL mRNA expression was determined by qRT-PCR. All samples were normalized to 18S rRNA and graphed as expression relative to BxPC3 (lane 9). n = 3, error bars  = 1 S.D. (B) Hypoxic SNAIL mRNA expression was graphed relative to hypoxic PHD3 expression as was determined in (Figure 4B). ρ = Pearson's correlation coefficient. Line represents best fit for the data.

To test this hypothesis, we subjected cells to 24 hours of normoxia or hypoxia and measured SNAIL and E-cadherin expression at the mRNA level. As expected, the cell lines with an epithelial morphology contained relatively high expression of E-cadherin mRNA and relatively low levels of SNAIL when compared to the more mesenchymal-like MiaPaca2 and Panc1 cells (**Figure 5A**). Furthermore, we found a nearly perfect negative correlation between PHD3 expression and SNAIL gene expression in these cell lines (r^2^ = .97) (**Figure 5B**). These results indicate that, like shRNA-mediated silencing of PHD3 in PDA cells, natural silencing of PHD3 in pancreatic cancer also appears to be associated with a mesenchymal-like phenotype at the level of gene expression.

To determine whether PHD3 expression could revert MiaPaca2 cells from their mesenchymal-like morphology back to a more epithelial phenotype, we created a stable PHD3-Wt-overexpressing MiaPaca2 cell line. We were able to achieve high levels of PHD3 protein expression in pooled populations of MiaPaca2, however PHD3 expression did not appear to affect cellular morphology, nor did it affect the expression of E-cadherin, which remained undetectable (data not shown). These findings indicate that restoration of PHD3 expression is not sufficient to restore an epithelial morphology in mesenchymal-appearing cancer cell lines.

Since PHD3 expression in PDA cells appears to correlate highly with E-cadherin expression, we asked whether stimuli that are known to downregulate E-cadherin in cancer cells (TGF-β or SNAIL overexpression) would also result in a downregulation of PHD3. Therefore, we attempted to stimulate an EMT-like process in BxPC3 cells by overexpressing SNAIL or stimulating with TGF-β. Although we did achieve high levels of stable SNAIL expression in these cells (**Figure S2**), we did not observe a significant decrease in PHD3 or E-cadherin mRNA expression or a change in cell morphology. Similarly, TGF-β had no effect on BxPC3 cell morphology (data not shown). Therefore, to more effectively determine the role of EMT on PHD3 silencing, we opted to use Madin-Darby Canine Kidney (MDCK) cells as a model system. This cell line is highly utilized for studies of EMT-like processes, as they are well known to significantly downregulate E-cadherin and undergo a stark morphological change following stimulation with TGF-β or overexpression of SNAIL.

### PHD3 expression correlates with E-cadherin expression in MDCK subpopulations

**Figure 6:**
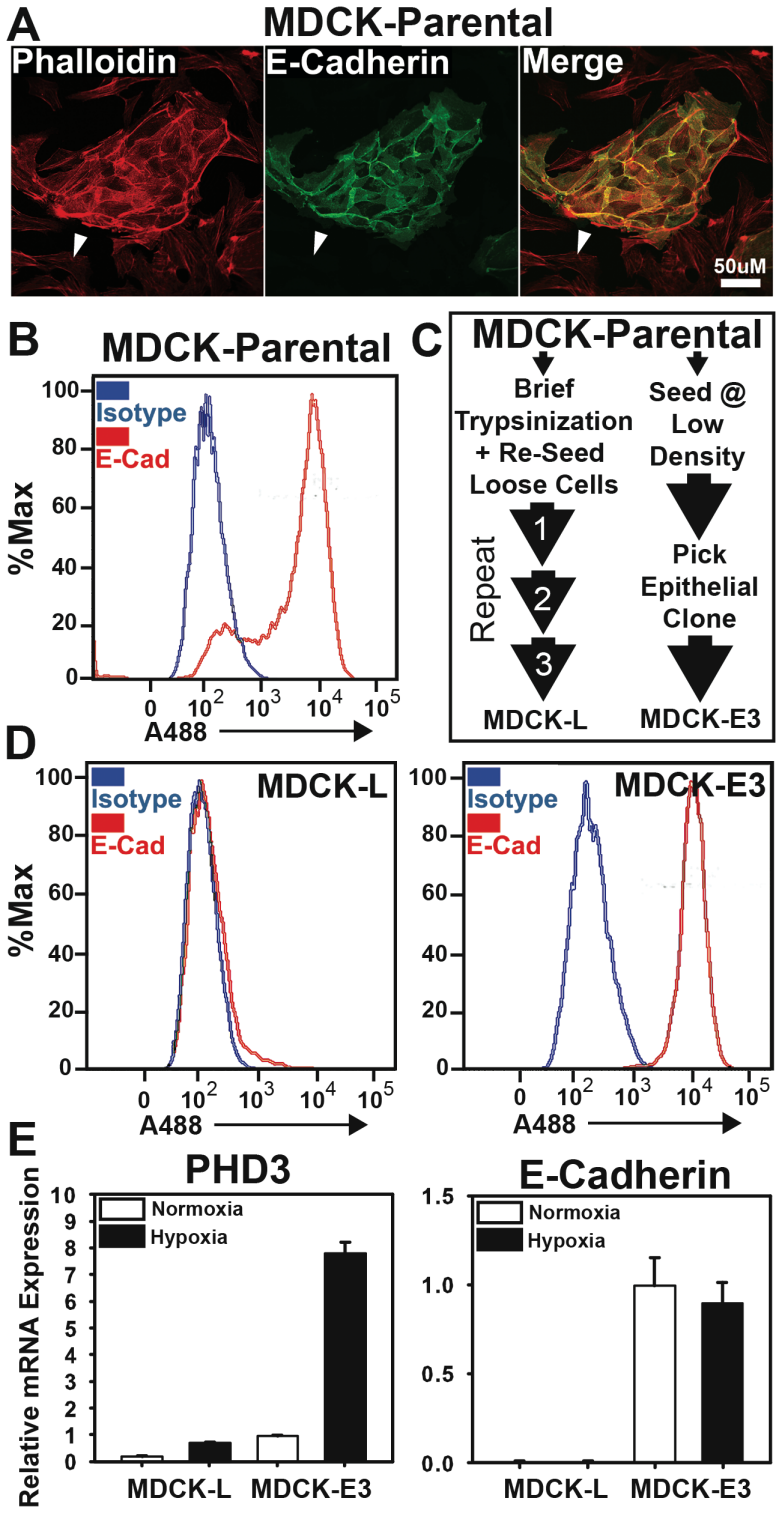
Flow cytometric analysis of E-cadherin in MDCK cells. (A) MDCK cells (parental population) were labeled with phalloidin (red) and anti-E-cadherin antibody (green) and visualized by confocal microscopy. Arrow indicates E-cadherin negative cells within the parental population. (B) Live MDCK cells (parental population) were labeled with primary anti-E-cadherin (red) or isotype-matched control antibody (blue), then with goat anti-mouse Alexa-fluor 488 (A488)-conjugated secondary antibody and analyzed by flow cytometry. (C) Flow chart for separation of mesenchymal and epithelial subpopulations of the MDCK parental cell line. (D) MDCK-L and MDCK-E3 cell lines were analyzed for surface E-cadherin expression by flow cytometry as done in (A). (E) MDCK-L and MDCK-E3 cells were subjected to normoxia (21% O_2_) or hypoxia (1% O_2_) for 24 hours. mRNA was harvested and PHD3 and E-cadherin expression was quantified by qRT-PCR. All data points represent the average of 3 biological replicates. Quantification of mRNA is set relative to MDCK-E3 samples at normoxia. Error bars  = +/− 1 S.D.

The Madin-Darby Canine Kidney (MDCK) cell line is a renal epithelial cell line developed by Madin and Darby in 1958. From this original parental line, several clones (namely MDCK-I and MDCK-II) have been described that vary in morphology as well as expression of E-cadherin [17]–[20]. To determine if our population of parental MDCK cells was homogeneous or heterogeneous with regard to E-cadherin expression, we first assayed the expression status of E-cadherin by both immunofluorescence and flow cytometry (**Figure 6A and B**). We found that approximately 20% of MDCK cells were present in a distinctly E-cadherin negative population. These E-cadherin negative cells appeared to have an elongated, spindle-shaped morphology. The remaining cells were E-cadherin positive and tended to form colonies of tightly connected cells. Using multiple rounds of brief trypsinization and collection of loosely adherent cells, we were able to isolate a subpopulation of cells that were nearly 100% E-cadherin negative. These cells were named MDCK-Loose (MDCK-L) (**Figure 6C**), and displayed a stable elongated, spindle-shaped morphology characteristic of mesenchymal cells. We also isolated an E-cadherin positive clone of MDCK cells by seeding the parental line at low density and picking a colony with a distinct epithelial morphology. This clone was named MDCK-Epithelial (MDCK-E3) (**Figure 6C**) and appeared to be a pure population of E-cadherin positive cells when analyzed by flow cytometry (**Figure 6D**). Using PCR amplification of genomic DNA, we further concluded that the MDCK-E3 and MDCK-L populations are of canine origin and not contaminants from another human cell line (**Figure S3**).

In order to determine the PHD3 expression status in MDCK-L and MDCK-E3 populations, we subjected them to 24 hours of normoxia or hypoxia followed by analysis of PHD3 expression by quantitative real-time PCR (qRT-PCR). We found that PHD3 mRNA was relatively highly expressed at normoxia in MDCK-E3 cells, and this expression was induced nearly 8-fold by hypoxia. In contrast, MDCK-L cells expressed significantly less PHD3 mRNA under normoxic and hypoxic conditions. In fact, hypoxic expression of PHD3 in the MDCK-L cells failed to reach normoxic MDCK-E3 levels (**Figure 6E**). Since PHD3 expression under hypoxia is driven by HIF, we considered that a blunted HIF-response in MDCK-L cells might explain this result. Surprisingly however, the HIF-responsive genes VEGF and CAIX were much more highly induced in the MDCK-L cells as compared to MDCK-E3 cells, whereas the HIF-responsive PHD2 was equally induced under hypoxia in both cells. These results strongly indicated that the reduction in PHD3 expression in the MDCK-L subpopulation is not due to a general defect in the hypoxia response pathway, but is specific to PHD3.

### Induction of EMT in MDCK-E3 results in PHD3 downregulation

**Figure 7:**
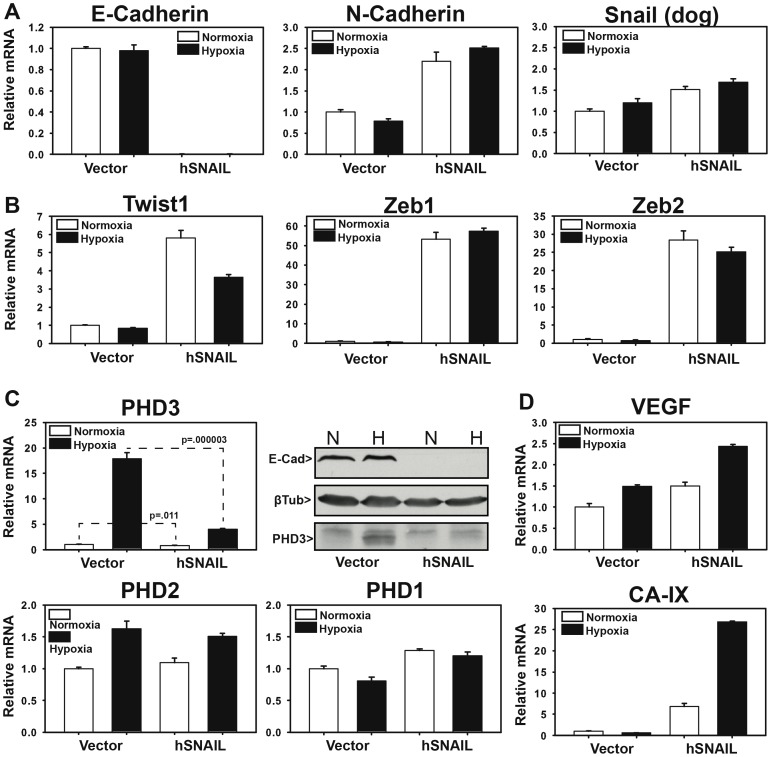
SNAIL-induced EMT in MDCKE3 Cells. (A–D). Stable pQCXIP-SNAIL (hSNAIL) or pQCXIP-vector (Vector)-expressing MDCK-E3 cells were subjected to normoxia (21% O_2_) or hypoxia (1% O_2_) for 24 hours. mRNA was harvested and subjected to qRT-PCR analysis for the indicated genes. All data points represent the average of 3 biological replicates. mRNA quantification is set relative to the Vector samples at normoxia. Error bars  = 1 S.D.

Using the MDCK-E3 clone, we first attempted to induce EMT by stable overexpression of human-SNAIL (MDCK-E3-hSNAIL). As expected, MDCK-E3-hSNAIL cells underwent a morphological change consistent with EMT, becoming spindle-shaped with a loss of cell-cell adherence. It is well known that morphological changes that occur during EMT are driven by upregulation of transcription factors such as SNAIL, Zeb1, Zeb2, and Twist. These transcription factors direct the downregulation of E-cadherin and a concurrent upregulation of N-cadherin, termed “cadherin switch”. As expected, when we stably overexpressed hSNAIL in MDCK-E3 cells, we saw significant downregulation of E-cadherin and upregulation of N-cadherin mRNA when compared to vector control cells. With regard to transcription factor expression, hSNAIL overexpression only mildly upregulated endogenous dog-SNAIL levels, whereas it significantly induced the expression of the other EMT transcriptional repressors Twist, Zeb1, and Zeb2 as compared to vector control (**Figure 7A and B**). To ensure that any apparent changes in gene expression after EMT or following exposure to hypoxia could not be accounted for by changes in the expression or our reference RNA (18S), we analyzed the Ct values of 18S rRNA for each sample. As expected, 18S rRNA proved to be expressed at consistent levels regardless of experimental condition (**Figure S4**). Overall, the above results demonstrate that MDCK-E3-hSNAIL cells undergo molecular and morphological changes consistent with EMT.

To determine whether PHD3 expression is downregulated by hSNAIL-mediated EMT induction, we compared PHD3 mRNA and protein levels in MDCK-E3-hSNAIL cells with PHD3 expression in vector control cells under normoxic and hypoxic conditions. We found that EMT-induction by hSNAIL resulted in significant reductions in both basal and hypoxia-inducible expression of PHD3 mRNA and protein. Interestingly, the expression of PHD1 and PHD2 were unaffected (**Figure 7C**). We also found that the expression of HIF-responsive VEGF and CA-IX mRNA were expressed at higher basal levels in cells that had undergone EMT, and, in the case of CA-IX appeared to become hyper-inducible by hypoxia following the induction of EMT (**Figure 7D**). This is similar to our observations in MDCK-L vs. MDCK-E3 cells described in the previous section.

Aside from overexpression of exogenous hSNAIL, TGF-β treatment has been reported to induce EMT in MDCK cells; therefore, we treated MDCK cells with 10 pg/ml TGF-β to see if we could replicate the PHD3 downregulation seen above. Like the hSNAIL overexpressing cells, TGF-β-treated cells underwent a stark morphological change consistent with EMT. TGF-β treatment also resulted in a similar change in EMT-related gene expression as did the hSNAIL overexpressing cells. This included a significant downregulation of basal and hypoxia-inducible PHD3 mRNA levels, with no significant effect on PHD1 or PHD2 expression (**Figure S5**).

Previously, we demonstrated that DNA methylation of the *PHD3* promoter is a mechanism of PHD3 silencing in human cancer cell lines [10]. Therefore, we investigated whether the promoter of *PHD3* becomes silenced in MDCK cells that have undergone SNAIL or TGF-β-induced EMT. We used bisulfite conversion of genomic DNA followed by Sanger sequencing of cloned PCR products from MDCK-E3, MDCK-L, TGFβ-treated MDCK and MDCK-E3-SNAIL cells. However, we did not detect any methylated CpGs in any of these samples, indicating that EMT-induced PHD3 downregulation in MDCK cells does not result in *PHD3* promoter DNA methylation (data not shown).

## Discussion

In the present study, we make the novel observation that PHD3 expression is tightly linked to epithelial and mesenchymal cell differentiation status. In epithelial cells, as well as cancer cell lines displaying an epithelial morphology in cell culture, PHD3 is very highly induced upon exposure to hypoxic conditions. On the other hand, we found that this hypoxia-inducible expression is significantly blunted to nearly undetectable levels in epithelial cells that have been induced to undergo EMT and in pancreatic cancer cells that have a mesenchymal morphology. Our finding that human fibroblasts do not express PHD3 mRNA or protein under normoxic or hypoxic conditions substantiates this data. These latter observations are supported by data from Wax *et al*., who noted a lack of PHD3 (SM-20) expression in 3T3 and rat 6 fibroblasts during their initial discovery and characterization of PHD3[21].

We believe that the association between PHD3 downregulation and mesenchymal differentiation status may be significant in the context of cancer, and could be related to the migratory capacity of cancer cells. When we knocked down PHD3 expression in BxPC3 cells, we observed a change in the connectivity of the cells to one another in culture, along with an increase in their migratory capacity. These results support observations made by Su *et al*. who reported that knockdown of PHD3 in pancreatic cancer cell lines resulted in an increase in cell invasion through Matrigel [9].

What is not immediately forthcoming in our study is why epithelial cells express high levels of PHD3, and why PHD3 might be downregulated during EMT and in fibroblasts. Considering our observation that the morphology of BxPC3 cells changes following knockdown of PHD3, we hypothesize that PHD3 may be involved in control of epithelial cell structure or polarity. This would support a model whereby PHD3 downregulation during EMT might be permissive for cytoskeletal structural changes in the cell that allow for EMT-induced cell motility.

A survey of the PHD3 literature identifies several pieces of data that support this hypothesis. First, a previous study has shown that PHD3 loss in the context of neuronal development results in increased axon growth and branching[22]. These are processes that involve similar molecular mechanisms as cell motility[23]. In another report, a murine knockout of PHD3 was shown to result in an exacerbated macrophage-mediated immune response to abdominal sepsis. A notable characteristic of the isolated *PHD3* ^-/-^ macrophages was an increase in their migratory capacity[8]. In addition to these data that are specific to PHD3, a general role for prolyl-hydroxylases in the regulation of cell motility can be found within studies of more primitive organisms. In *D. discoideum* (slime mold), oxygen gradients are utilized to direct cell motility. This process may be regulated through O_2_ sensing by a prolyl-4-hydroxylase that is evolutionarily related to mammalian PHD1-3 enzymes[17], [19], [20]. Overall, these data are highly suggestive of a role for prolyl-hydroxylation, potentially through a PHD3-mediated mechanism, in the maintenance of cell structure, polarity and migration. Nonetheless, this hypothesis needs to be more carefully analyzed in future studies.

Another potential role for PHD3 downregulation during EMT might be evasion of apoptosis. One characteristic of cells that have undergone EMT is resistance to apoptosis[24]. Numerous studies have indicated PHD3 as a pro-apoptotic protein[9], [25]–[33]. Therefore, downregulation of PHD3 during EMT may contribute to the apoptosis-resistant phenotype of these cells. Furthermore, the potential link between PHD3 and cell motility could point to a role for PHD3 in anoikis, a form of apoptosis linked to cell adhesion[34].

During this study, we attempted to determine the mechanism by which EMT induction results in suppression of PHD3 expression. Previous studies in our laboratory have identified DNA methylation as a mechanism of PHD3 silencing in some cancer cell lines. Therefore we initially hypothesized that PHD3 downregulation following EMT induction by TGF-β treatment and *SNAIL* overexpression might involve DNA methylation. This does not appear to be the case, as we did not detect evidence of *PHD3* promoter methylation in MDCK cells that had undergone EMT. Interestingly though, inspection of the UCSC genome browser conserved transcription factor binding site track (TFBS Conserved, (http://genome.ucsc.edu/)) predicts a highly conserved binding site for Zeb1 (AREB6) just downstream of *PHD3* exon 1 (**Figure S6**)[35]. This suggests that *PHD3* may be a Zeb1 target gene. Indeed, both TGF-β treatment and *SNAIL* overexpression led to upregulation of Zeb1 in our MDCK cells, and subsequently resulted in the induction of EMT. We attempted chromatin immunoprecipitation (ChIP) multiple times for Zeb1 on the PHD3 promoter, however we were unable to overcome technical limitations and produce reliable data. Therefore, Zeb1-mediated suppression of *PHD3* transcription remains a potential mechanism that needs to be investigated in future studies.

Aside from transcriptional repression by Zeb1 or other family members, another potential regulator of PHD3 expression during EMT is microRNA. In cardiomyocytes, PHD3 expression is known to be suppressed by a microRNA-mediated mechanism [36]. Furthermore, the PHD3 3′-untranslated region (UTR) contains a conserved miR-9 binding site as predicted by Targetscan.org (**See Figure S7**). This site is also present in the E-cadherin 3′-UTR and has been shown to be involved in E-cadherin downregulation during breast cancer metastasis [37]. Further studies will be needed to determine if Zeb1 and/or microRNAs are also involved in suppressing PHD3 expression in cells.

Overall, our data add to the current knowledge regarding the relationship between oxygen sensing pathways and EMT/cell motility. We believe that PHD3 expression in epithelial cells may normally play a functional role that opposes cell motility. In hypoxic conditions, epithelial cells may upregulate PHD3 in order to maintain proper epithelial organization. This might be attained through PHD3-mediated hydroxylation of a yet-to-be identified PHD3 target protein involved in cytoskeletal arrangement. Under certain hypoxic conditions, such as tissue damage, epithelial cells may need to mobilize to promote healing. In this case, downregulation of PHD3 expression following hypoxia and/or cytokine-mediated EMT may have evolved to disengage PHD3-mediated hydroxylation, and allow for cell cytoskeletal changes necessary for cell motility. This hypothesis is supported by our evidence that PHD3 knockdown in BxPC3 cells results in a defect in cell-cell attachments, and other reports of morphology and migratory changes following PHD3 loss in other cell types[8], [9].

Another question that is still unresolved is that of hypoxia-induced EMT. A large body of evidence shows that exposure of epithelial cells to hypoxia can, in some cases, lead to the induction of EMT[3]. In this study we attempted to induce EMT in cells by simple exposure to hypoxic conditions. Although the migratory speed of BxPC3-Vec or BxPC3-Wt cells was modestly but significantly increased in hypoxic conditions, we were not able to appreciate any significant morphological changes in these cells in culture that would be consistent with EMT induction. Interestingly though, we found that knockdown of PHD3 in BxPC3 cells resulted in an increase in migratory capacity that was not further enhanced by hypoxia. One explanation for these findings is that PHD3 expression acts as a “cell-motility brake”, limiting the extent of the hypoxia-induced motility response in epithelial cells.

Further studies will be needed to support the aforementioned hypotheses. These future studies might include quantification of PHD3 expression in primary vs. metastatic cancer cells. Currently, the availability of pancreatic cancer specimens for this type of study is a limiting factor, as metastatic lesions are not surgically removed. Furthermore, EMT is a transient process, and metastatic cells do not typically retain the EMT/pro-migratory phenotype upon metastatic colonization. Therefore, studies aimed at determining PHD3 expression status should be aimed at circulating tumor cells, or individual migrating cells within the primary tumor.

Future studies should also aim to identify other targets of PHD3 expression. The number of validated PHD3 targets is increasing, and include Pyruvate-kinase M2 isoform, beta2-adrenergic receptor, hCLK2, the HIF-α proteins and numerous other potential protein targets representing a heterogenous array of signaling pathways[4], [32], [38]–[40]. In fact, a simple search of proteins containing the hydroxylation consensus sequence (LXXLAP) using scansite3.mit.edu reveals several hundred potential PHD target proteins (**Table S2**)[41]. Of interest, five Rho guanine exchange factors (RhoGEFs), a Cdc42 GTPase activating protein (Cdc42GAP), as well as many other F-actin interacting proteins contain LXXLAP motifs (**Table S3**).

PHD3 is rapidly evolving as a protein with many functions[4]. Our data add a new layer of understanding to the regulation of PHD3 expression, and hint at a novel role for PHD3 expression in EMT and cell migration. Ultimately, we hope these data, as well as future research on PHD3, will aid in the understanding of how oxygen affects cell cancer metastasis and other physiological processes that depend on EMT and cell migration.

## Supporting Information

Figure S1 E-cadherin surface expression is not effected by stable PHD3 overexpression of knockdown in BxPC3 cells. BxPC3 cells stably overexpressing PHD3-Wt (BxPC3-Wt), Vector (BxPC3-Vec), or PHD3 knockdown shRNA construct #48 (BxPC3-KD) were assayed for surface E-cadherin expression using flow cytometry as described in the methods section. A) Flow histograms are depicted with red lines representing cells labeled isotyped-matched control antibody and FITC-conjugated secondary antibody. Blue lines indicate cells labeled with E-cadherin primary antibody and FITC-conjugated secondary antibody. MiaPaca2 cells were used as a negative control as they are known to be E-cadherin negative B) The % of cells gated as E-cadherin positive in the isotype mached control groups (Iso) and the E-cadherin antibody groups (E) are graphed. Gates were set at approximately 10^3^.
(PDF)

Figure S2 SNAIL overexpression in BxPC3 cells. BxPC3 parental cells, and BxPC3 containing stable expression of Vector (Vec) or SNAIL were exposed to normoxia (21% O_2_) or hypoxia (1% O_2_) for 24 hours. mRNA was harvested and subjected to qRT-PCR analysis for the indicated genes. mRNA values are graphed relative to BxPC3 samples under normoxic conditions. n = 1.
(PDF)

Figure S3 MDCK subpopulations are of dog origin and are not contaminants. The indicated human cell lines MDA-MB-435 (MB-435) and BxPC3, along with the dog cell line MDCK II, and the cell lines MDCK-E3 and MDCK-L (which we derived from the MDCK parental cell line) were harvested for genomic DNA (gDNA). PCR primers were designed to a homologous region in the human and dog genome that contains a small 310 bp deletion in the middle of the amplicon only in the dog. Thus, dog gDNA can be discriminated from human by a smaller amplicon size.
(PDF)

Figure S4 18S rRNA remains stable regardless of treatment. Ct values of 18S rRNA are plotted for each sample. This data was extracted from qRT-PCR data for samples in Figure 7 and contains 3 replicates from each sample. Error Bars  = 1 S.D..
(PDF)

Figure S5 TGF-β induced EMT in MDCK Cells. (A–D). Parental MDCK cells were treated with 10 pM TGF-β and then subjected to normoxia (21% O_2_) or hypoxia (1% O_2_) for 24 hours. mRNA and protein (top right only) was harvested and subjected to qRT-PCR and western blot (top right only) analysis for the indicated genes. All data points represent the average of 3 biological replicates. mRNA quantification is set relative to the MDCK control samples at normoxia. Error bars  = 1 S.D..
(PDF)

Figure S6 Predicted transcription factor binding sites in the *PHD3* promoter. The UCSC genome browser (GFCh37/hg19) HMR Conserved Transcription Factor Binding Site “TFBS Conserved” track was used to predict transcription factor binding sites on the *PHD3* promoter (http://genome.ucsc.edu/)[35]. A Z-score of 2.1 was used.
(PDF)

Figure S7 Predicted miRNA binding sites on the *PHD3* 3′UTR. Human “EGLN3” (PHD3) was queried on Targetscan.org (release 6.2). A modified screenshot of the output is depicted.
(PDF)

Table S1 Primers used in this study. A list of SYBR Green primers and primer sets used for bisulfite sequencing of the dog PHD3 promoter (meth-PHD3) are listed. F = Forward, R = Reverse. For methylation-specific primers, nested PCR was used with outer primers used in the first reaction, followed by inner primers.
(XLSX)

Table S2 List of human genes encoding for proteins that contain an LXXLAP motif. Scansite3.mit.edu was used to search for proteins in the Human Ensemble database containing the sequence pattern L-X-X-L-A-P.[41] Protein IDs were converted to Gene IDs, which were uploaded as a gene list into DAVID (http://david.abcc.ncifcrf.gov/) [42], [43]. The complete output list from DAVID is shown in this table.
(XLSX)

Table S3 List of LXXLAP-containing genes that are functionally related to “Cytoskeleton”, “Cell Projection” and “Cell Adhesion”. The DAVID functional annotation tool (http://david.abcc.ncifcrf.gov/) was used to determine which LXXLAP-containing proteins might be related processes involving cell adhesion or migration[42], [43]. Genes with DAVID annotations in the functional categories of “Cytoskeleton”, “Cell Projection” and “Cell Adhesion” are shown in this table.
(XLSX)
