# Supplementary figures and images for: Prolyl-4-Hydroxylase 3 (PHD3) Expression Is Downregulated during Epithelial-to-Mesenchymal Transition
Source: PLoS One. 2013 Dec 18;8(12):e83021. doi: 10.1371/journal.pone.0083021 (PMC3867438; doi:10.1371/journal.pone.0083021)

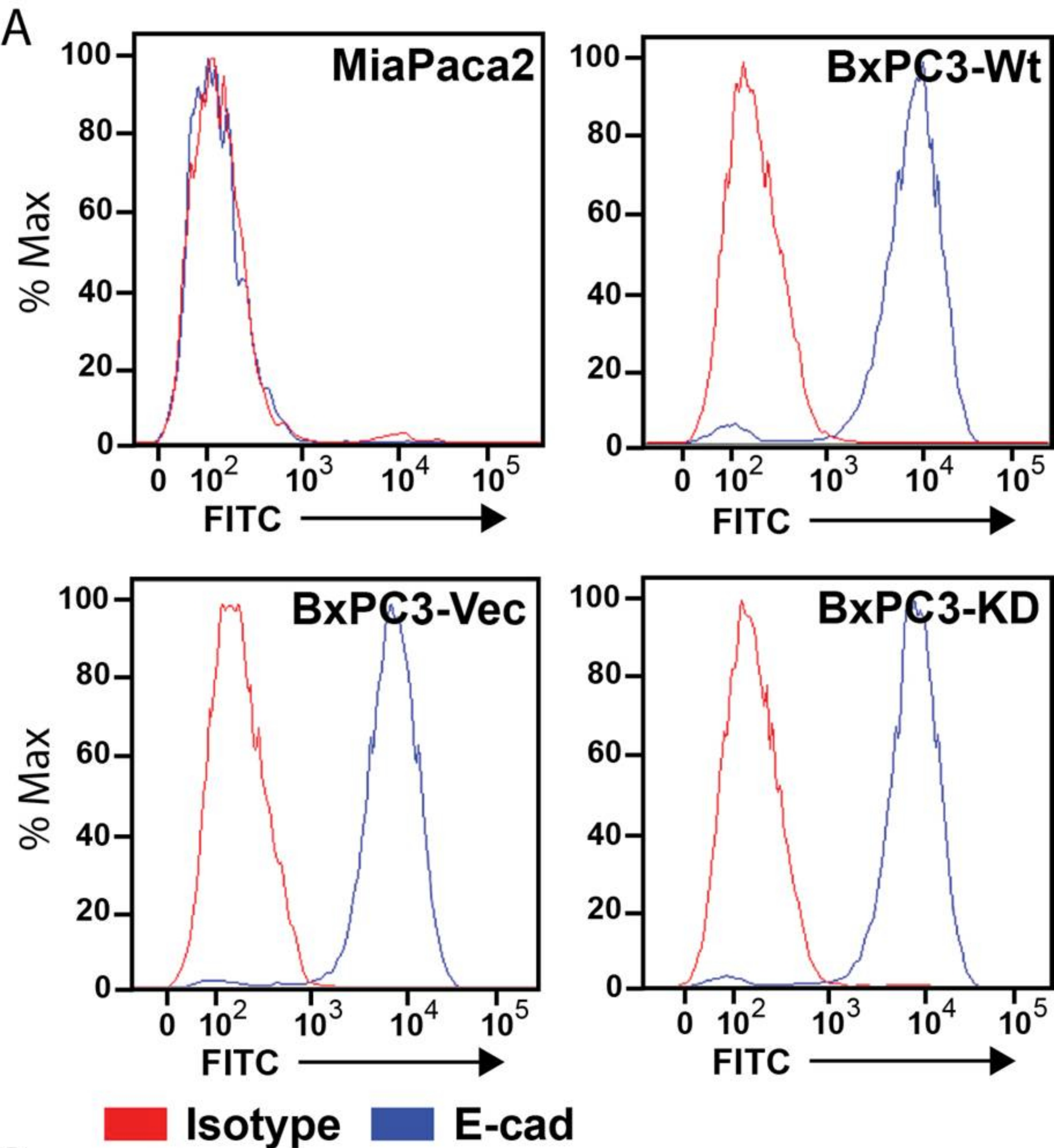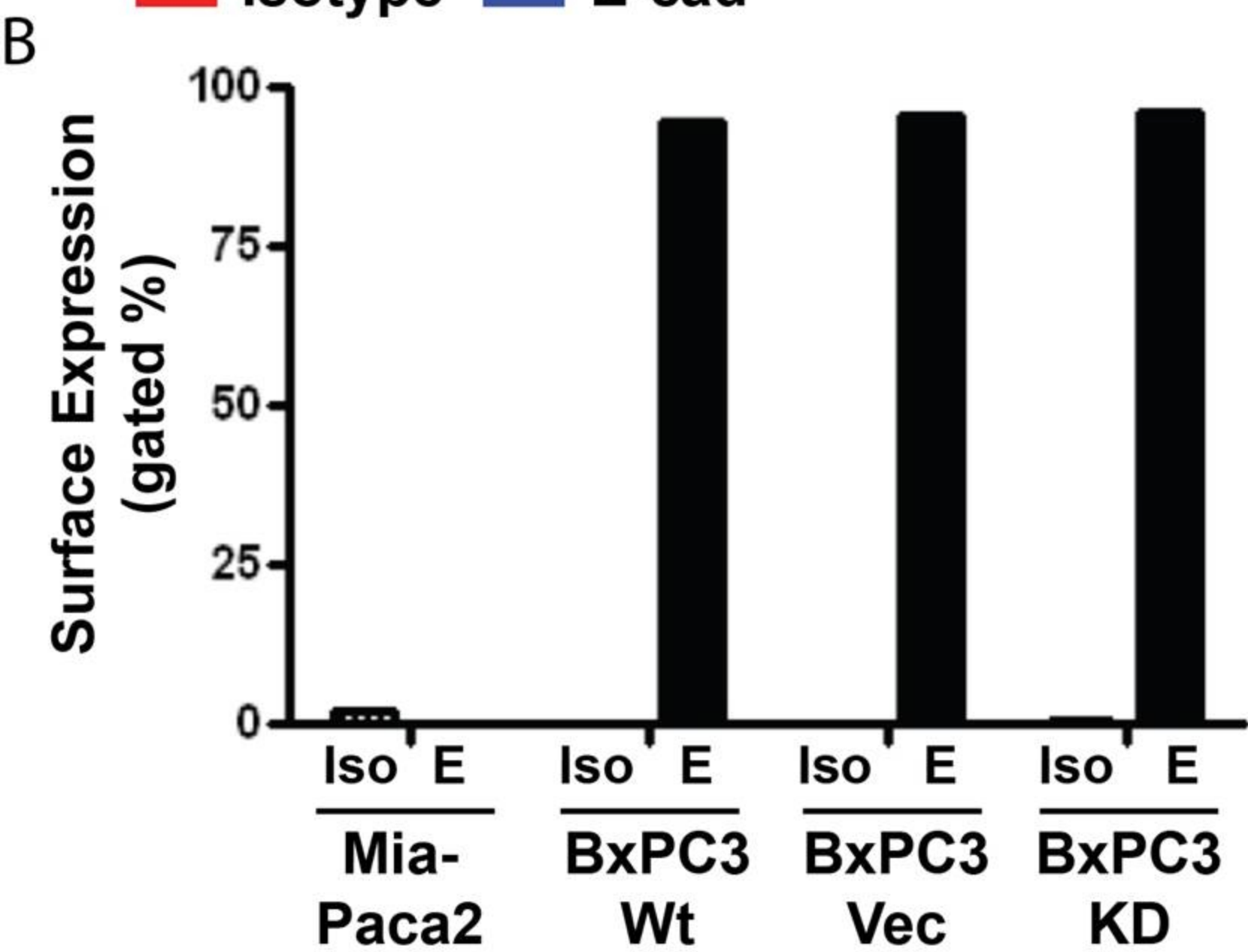

Supplement: Figure S1 — E-cadherin surface expression is not effected by stable PHD3 overexpression of knockdown in BxPC3 cells. BxPC3 cells stably overexpressing PHD3-Wt (BxPC3-Wt), Vector (BxPC3-Vec), or PHD3 knockdown shRNA construct #48 (BxPC3-KD) were assayed for surface E-cadherin expression using flow cytometry as described in the methods section. A) Flow histograms are depicted with red lines representing cells labeled isotyped-matched control antibody and FITC-conjugated secondary antibody. Blue lines indicate cells labeled with E-cadherin primary antibody and FITC-conjugated secondary antibody. MiaPaca2 cells were used as a negative control as they are known to be E-cadherin negative B) The % of cells gated as E-cadherin positive in the isotype mached control groups (Iso) and the E-cadherin antibody groups (E) are graphed. Gates were set at approximately 103. (PDF) [file pone.0083021.s001.pdf]

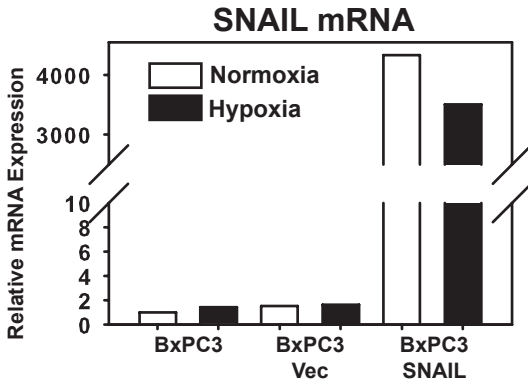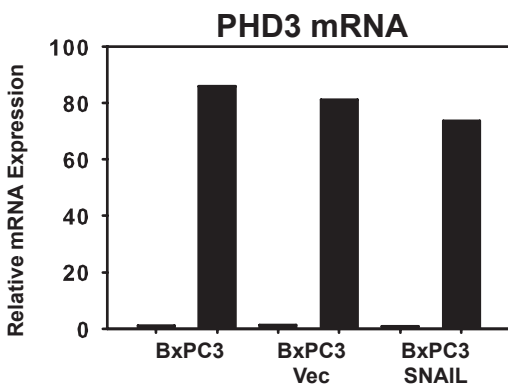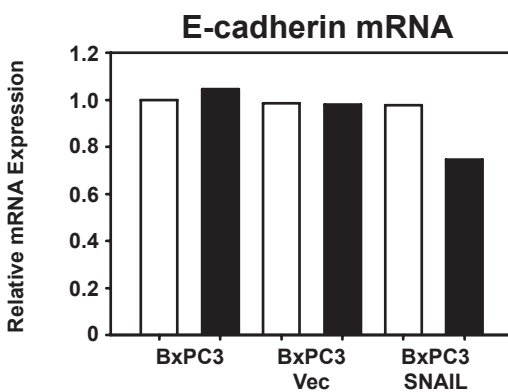

Supplement: Figure S2 — SNAIL overexpression in BxPC3 cells. BxPC3 parental cells, and BxPC3 containing stable expression of Vector (Vec) or SNAIL were exposed to normoxia (21% O2) or hypoxia (1% O2) for 24 hours. mRNA was harvested and subjected to qRT-PCR analysis for the indicated genes. mRNA values are graphed relative to BxPC3 samples under normoxic conditions. n = 1. (PDF) [file pone.0083021.s002.pdf]

## 18S rRNA

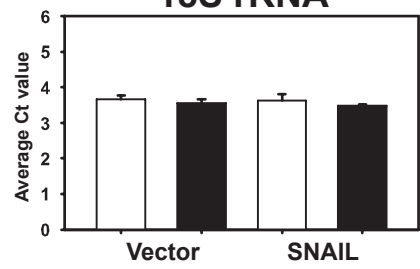

Supplement: Figure S4 — 18S rRNA remains stable regardless of treatment. Ct values of 18S rRNA are plotted for each sample. This data was extracted from qRT-PCR data for samples in Figure 7 and contains 3 replicates from each sample. Error Bars = 1 S.D.. (PDF) [file pone.0083021.s004.pdf]

### PHD3

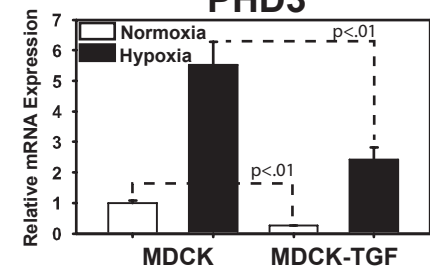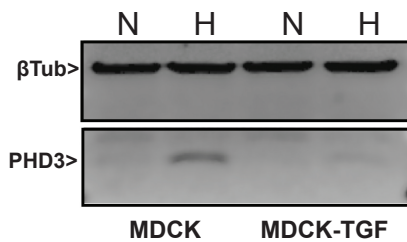

### PHD2

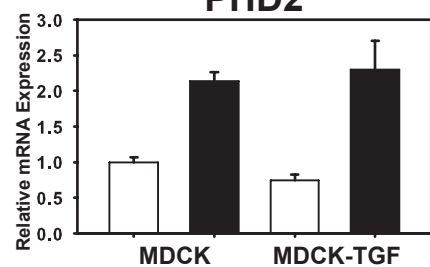

### PHD1

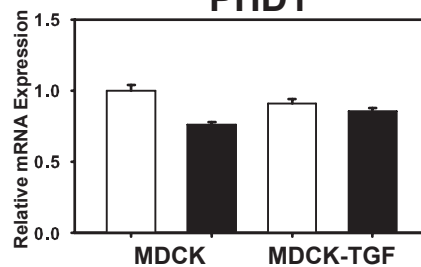

### E-Cadherin

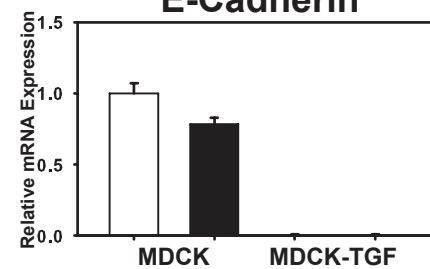

### N-Cadherin

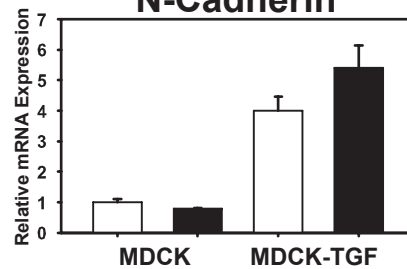

### Snail

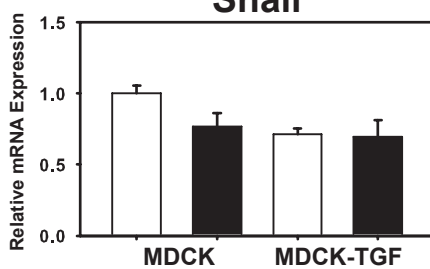

### Zeb1

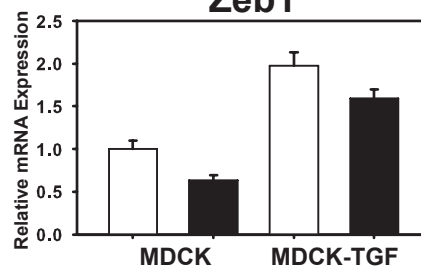

### CA-IX

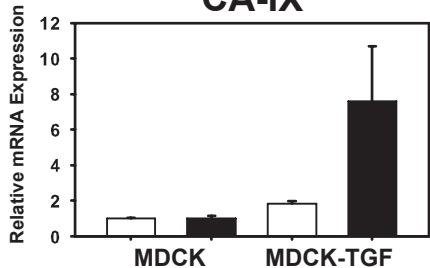

### Vegf

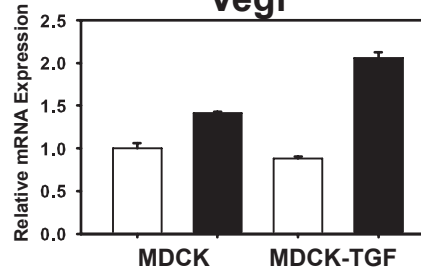

Supplement: Figure S5 — TGF-β induced EMT in MDCK Cells. (A–D). Parental MDCK cells were treated with 10 pM TGF-β and then subjected to normoxia (21% O2) or hypoxia (1% O2) for 24 hours. mRNA and protein (top right only) was harvested and subjected to qRT-PCR and western blot (top right only) analysis for the indicated genes. All data points represent the average of 3 biological replicates. mRNA quantification is set relative to the MDCK control samples at normoxia. Error bars = 1 S.D.. (PDF) [file pone.0083021.s005.pdf]

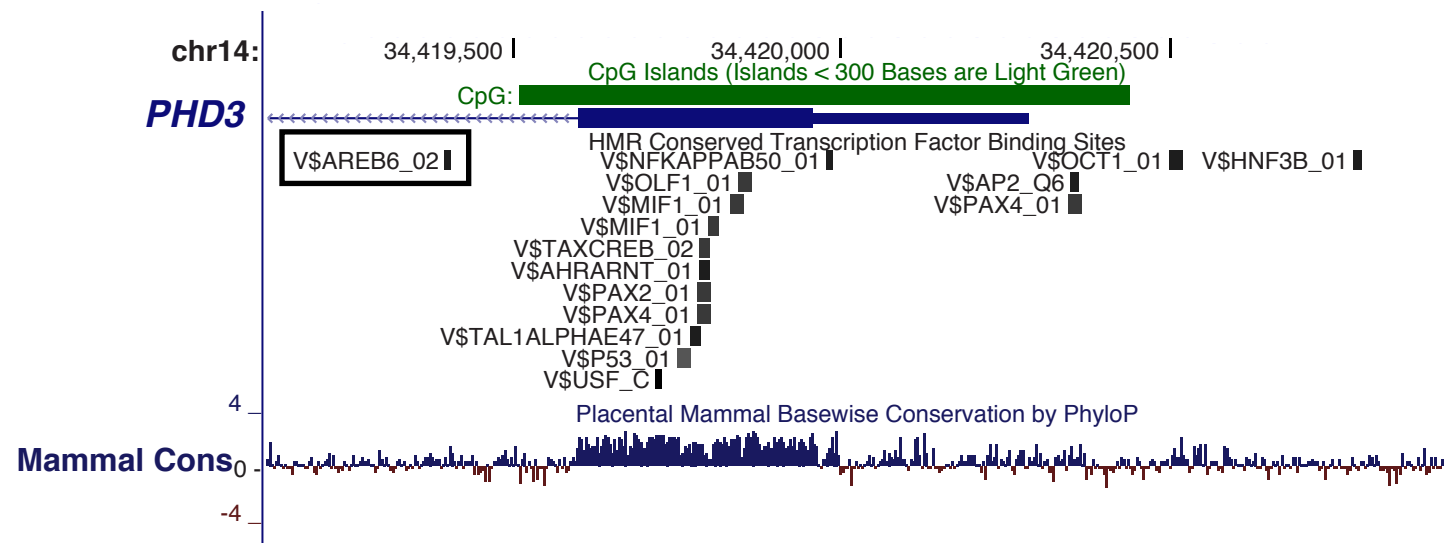

Supplement: Figure S6 — Predicted transcription factor binding sites in the PHD3 promoter. The UCSC genome browser (GFCh37/hg19) HMR Conserved Transcription Factor Binding Site “TFBS Conserved” track was used to predict transcription factor binding sites on the PHD3 promoter (http://genome.ucsc.edu/)[35]. A Z-score of 2.1 was used. (PDF) [file pone.0083021.s006.pdf]
