# Supplementary material for: Prolyl-4-Hydroxylase 3 (PHD3) Expression Is Downregulated during Epithelial-to-Mesenchymal Transition
Source: PLoS One. 2013 Dec 18;8(12):e83021. doi: 10.1371/journal.pone.0083021 (PMC3867438; doi:10.1371/journal.pone.0083021)
Supplement: Figure S3 — MDCK subpopulations are of dog origin and are not contaminants. The indicated human cell lines MDA-MB-435 (MB-435) and BxPC3, along with the dog cell line MDCK II, and the cell lines MDCK-E3 and MDCK-L (which we derived from the MDCK parental cell line) were harvested for genomic DNA (gDNA). PCR primers were designed to a homologous region in the human and dog genome that contains a small 310 bp deletion in the middle of the amplicon only in the dog. Thus, dog gDNA can be discriminated from human by a smaller amplicon size. (PDF) [file pone.0083021.s003.pdf]

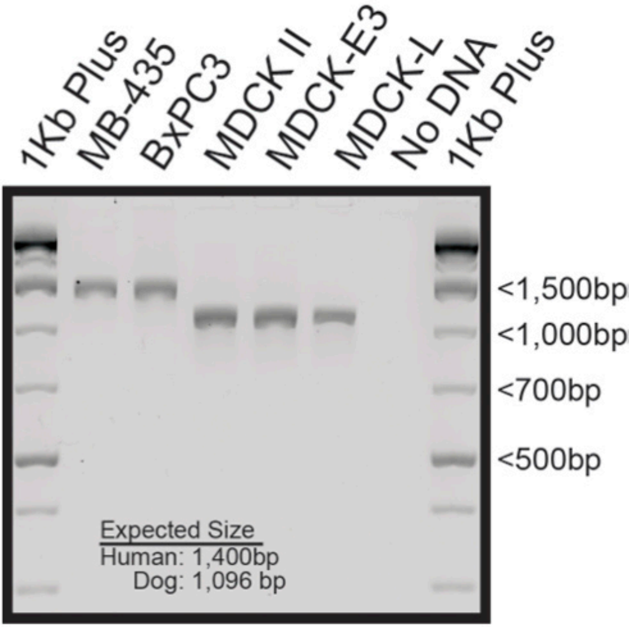

Fwd: TTCACCAAAGTTGAATCAGAAGA
Rev: TGACACCTACCTCAGGTGAACTT
