# Supplementary material for: Prolyl-4-Hydroxylase 3 (PHD3) Expression Is Downregulated during Epithelial-to-Mesenchymal Transition
Source: PLoS One. 2013 Dec 18;8(12):e83021. doi: 10.1371/journal.pone.0083021 (PMC3867438; doi:10.1371/journal.pone.0083021)
Supplement: Figure S7 — Predicted miRNA binding sites on the PHD3 3′UTR. Human “EGLN3” (PHD3) was queried on Targetscan.org (release 6.2). A modified screenshot of the output is depicted. (PDF) [file pone.0083021.s007.pdf]

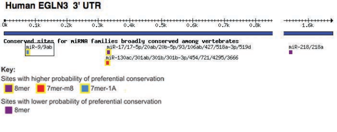
Human EGLN3 3' UTR
0k
0.1k
0.2k
0.3k
0.4k
0.5k
0.6k
0.7k
0.8k
1.6k
Conserved sites for miRNA families broadly conserved among vertebrates
miR-9/9ab
miR-17/17-5p/20ab/20b-5p/93/106ab/427/518a-3p/519d
miR-130ac/301ab/301b/301b-3p/454/721/4295/3666
miR-218/218a
Key:
Sites with higher probability of preferential conservation
8mer
7mer-m8
7mer-1A
Sites with lower probability of preferential conservation
8mer
